# Morphological Changes of Gingiva in Streptozotocin Diabetic Rats

**DOI:** 10.1155/2009/725628

**Published:** 2009-01-27

**Authors:** C. Tesseromatis, A. Kotsiou, H. Parara, E. Vairaktaris, M. Tsamouri

**Affiliations:** ^1^Department of Pharmacology, Medical School, University of Athens, Mikras Assias 75, 11527 Goudi, Greece; ^2^Pharmacy Department, Aretaeion University Hospital, Vassilis Sophias 76, 11528 Athens, Greece; ^3^Department of Craniofacial Surgery, Medical School, University of Athens, Mikras Asias 75, 11527 Goudi, Greece; ^4^Department of Pathology, Red Cross Hospital, Red Cross Street, 11526 Athens, Greece

## Abstract

Gingivitis and periodontitis are chronic bacterial diseases of the underlying and surrounding tooth tissues. Diabetes mellitus is responsible for tooth deprivation both by decay and periodontal disease. The streptozotocin-induced diabetes results in a diabetic status in experimental animals similar to that observed in diabetes patients. The aim of the study was to investigate the relationship between the gingival lesions and the microangiopathy changes in streptozotocin-induced diabetes mellitus. Forty male Wistar rats were divided into two groups (control and experimental). Diabetes mellitus was induced by 45 mg/kg IV streptozotocin. The histological investigation of the marginal gingival and the relevant gingival papilla showed inflammation of the lamina propria and the squamous epithelium as well as marked thickness of the arteriole in the diabetic group, but no changes were observed in the control group. The results suggested a probable application of a routine gingival histological investigation in diabetic patients in order to control the progress of disease complications. It may be concluded that histological gingival investigation can be used as a routine assay for the control of the diabetic disease and prevention of its complications.

## 1. Introduction

Gingivitis and periodontitis are chronic bacterial diseases of the underlying and surrounding tooth tissues. The initial factor in the development of the periodontal disease is the host response and its defense capacity to the microbial stimuli. Systemic factors modify all forms of gingivitis and periodontitis through their effect on the physiological immune and inflammatory defense. It has been reported [1] that nerve fibers are involved in the neurogenic inflammation induced by mechanical or chemical irritations in the gingival and the underlying tissues. It has also been suggested that in diabetes mellitus the unmyelinated small diameter fibers are impaired as a result of diabetic neuropathy. Furthermore, many studies have been focused in the impairment of bone mass, occurring in diabetes mellitus. Microangiopathy at the bone tissue was suggested as a possible cause of diabetic osteopenia [2]. Diabetes can have an impact on the bone through multiple pathways, some with contradictory effects, including obesity, changes in insulin levels, higher concentrations of advanced glycation end products in collagen, hypercalciuria associated with glycosuria, reduced renal function, lower insulin-like growth factor-I, microangiopathy, and inflammation [3].

Furthermore, diabetes mellitus is responsible for tooth deprivation both by decay and periodontal disease. Systemic diseases are associated with a higher experience of caries, a high ratio of decayed-to-present teeth, and more gingival and periodontal problems. Patients with high blood pressure, osteoporosis or diabetes mellitus tended to have poorer gingival or periodontal conditions, fewer teeth, and higher risk of edentulousness [4]. In addition, it was found that a larger number of oral *streptococci* adhered to the tooth surfaces are observed in nonobese diabetogenic mice that spontaneously develop insulin-dependent diabetes mellitus [5].

The aim of the study was to investigate the relationship between the development of gingival lesions and the presence of microangiopathy in streptozotocin-induced diabetes mellitus.

## 2. Material and Methods

Forty male Wistar rats of average body weight of 200 g were divided into groups A (experimental *n* = 20) and B (control, *n* = 10). They were housed five per cage at a constant room temperature (22 ± 1°C) under a 12-hour light/12-hour dark (light period 00.8–20.00 hours) cycle. Food and water were provided ad libitum. Animals were cared for accordance with the principles of the “Guide for the Care and Use of Experimental Animals” [6]. The animals of group A were injected once IV with streptozotocin 45 mg/kg in the jugular interna. The duration of the experiment was 90 days. The blood glucose levels were estimated every week with wash-off strips (Dextrostix, Ames Division, Miles Laboratories, Rexdale, Ontario, Canada) in blood obtained from the tail vessels. In parallel, glucose was also qualitatively assayed in urine with urineteststrips (Glukotest Accu-test Roche). The animals' body weight as well as their food intake was determined. The animals were sacrificed by decapitation, and gingiva specimens obtained from the incisor area of the mandible were washed with water and then conserved in 10% formalin solution for further histological examination with the light microscope. The histological slices of gingival specimens were stained with haematoxylin-eosin by light microscope. The values were expressed as mean ± standard deviation (m ± SD). The statistical analysis was performed by Student's *t*-test and by *x* ^2^ analysis. *P* < .05 was considered as an acceptable level of significance.

## 3. Results

The induction of diabetes mellitus was assessed the day after streptozotocin injection by evaluating clinical symptoms such as frequent urination, increased appetite, and weight loss. In comparison to the control group, the experimental animals exerted a hyperphagia accompanied with an increased daily food consumption. Furthermore, the streptozotocin animals had increased serum glucose and increased glycosylated haemoglobin (Hb A1c) levels. The severity of diabetes was indicated by the statistically significantly decreased body weight in the experimental animals in comparison to controls *P* < ,001 (Table 1). The quantity of daily food intake was increased in diabetic animals compared to controls. Blood glucose levels were significantly increased in the experimental group (Table 1). The levels of Hb A1c were lower in the control compared to the experimental group. The urination of the experimental group was frequent and the shavings of the cages needed to be changed twice daily.

The experimental animals had a mortality of 10% during the experimental period, while all the control animals remained alive until the end of the experimental procedure.

The histological findings of the experimental group were as follows. Through haematoxylin-eosin stain, the biopsies from the marginal gingivae and the gingival papillae from the incisor area showed inflammation of the lamina propria, formation of new vessels with various wall thicknesses, and hyperaemia. This alteration was localized in the internal site of the gingiva, which was in contact with the tooth surface.

**Figure 1:**
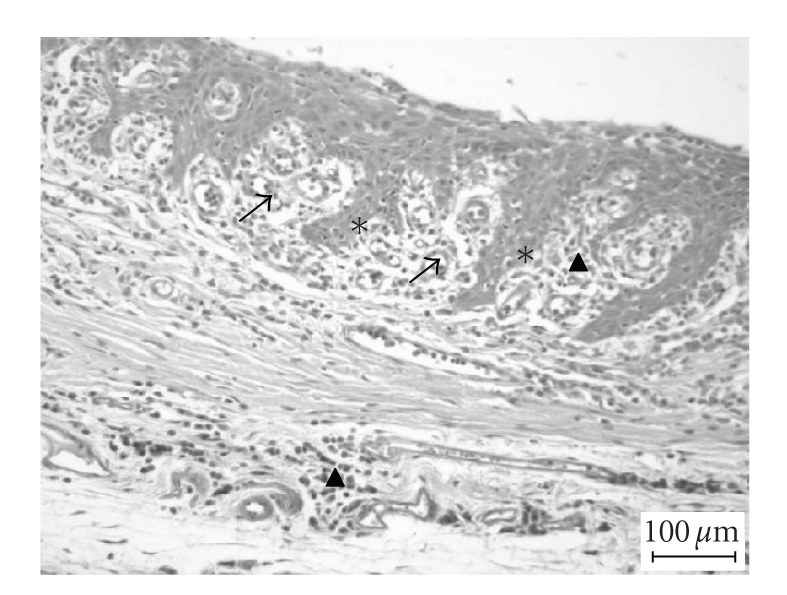
(▴) Focal perivascular and diffuse inflammation of the lamina propria, (↑) neo-angiogenesis, and (∗) hyperplasia of the squamous epithelium.

**Figure 2:**
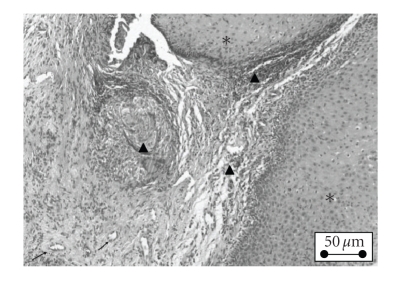
(∗) Hyperplasia of the squamous epithelium, (▴) diffuse and perivascular inflammation of the lamina propria, and (↑) thickening of the wall of some arterioles.

The gingival specimens obtained from the molar area of the mandible of the buccal and lingual sites showed moderate to high hyperceratosis and mild inflammation. All the experimental animals showed moderate-to-severe angiitis, which was not observed in the control animals (see Table 2, Figures 1 and 2).

## 4. Discussion

Recent drastic increase in diabetic population poses serious problems in both health sciences and socioeconomic services. The recent drastic increase in the diabetic population poses serious problems in both health sciences and socioeconomic services. The most important issue in the clinical management of diabetic patients is the chronic complications that contribute to a high morbidity and mortality [7, 8].

Hyperglycaemia, as a common feature of diabetes mellitus, is a cause of different pathogenic mechanisms influencing endothelial function.

Diabetes mellitus results in the development of large and micro-vessel damage. A series of changes occur independently of the presence of atherosclerosis. The abnormalities include accumulation of PAS-positive material, laminin, fibronectin, type IV collagen, and connective tissue with lack of acid mucopolysaccharides, and deposition of calcium. It is of particular interest that accumulation of PAS-positive material and lack of acid mucopolysaccharides are recognized as the histological markers of diabetic microangiopathy [2, 8–12].

In this study, the administration of streptozotocin in rats induced a diabetic status in the experimental animals similar to that observed in diabetic patients, as shown by the manifested clinical symptoms in accordance with previously reported literature [2, 13, 14].

The changes observed in bed vasculature concerned macro- and microangiopathy. The extention of microangiopathy is known to impair the function of various organs and systems and common problems are manifested as retinopathy, nephropathy, and neuropathy [15]. Periodontal disease is considered as the sixth complication of diabetes mellitus. It has long been observed that diabetic patients have greater tooth loss due to periodontal disease than nondiabetics of comparable age [16]. The severity of the periodontal disease is absolutely influenced by the degree of diabetic status [17]. The phenomenon may be due to the high tissue glucose concentration, as well as to the presence of metabolic products of the impaired glucose metabolism and it is recognized histologically as enlarged vessel wall with a narrowing of vessel lumen diameter. This process induces disabilities of vessel wall and, in general, leads to abnormal vasculature [12, 18]. Our results are in agreement with those of other investigators, who reported narrowing of vessel lumen diameter in diabetic subjects as observed by the increased uptake of PAS deposition in the vessel walls [9].

These changes can be aggravated through inflammatory cell infiltration that occurs after one week of steptozotocin treatment in the gingivomucosal tissue as reported by Fehér et al. [1]. Despite multiple and long-term studies, the pathophysiology of diabetic microangiopathy and its pathogenesis are not fully elucidated. Under chronic hyperglycemia, early stimuli elicit adaptive reactions of tissues showing acute inflammatory processes of vessel walls and changes of microangiopathy. The impaired glucose metabolism is recognized histologically as enlarged vessel wall with a narrowing of vessel lumen diameter. This situation in dental care can be recognized by delay healing of tooth extraction sockets, periradical lesions, and periodontal disease [4, 19, 20].

In addition, it has been suggested that the unmyelinated small-diameter afferent nociceptive C-fibers are impaired in diabetes mellitus, which indicates that in the streptosotocin-induced diabetic rat, gingivomucosal tissue is the prerequisite for neurogenic inflammation induced by mechanical or chemical irritations causing a pronounced vessel permeability. Furthermore, diabetic changes may be accompanied by decreased collagen production in rat periodontal tissue [21].

This process induces disabilities of vessel wall such as narrowed vessel calibre and in general leads to the formation of an abnormal vasculature [12, 19]. Since the ability of the diabetic's circulation to distribute blood is affected, especially during increased blood flow causing severe disturbances, the surrounding and underlying tooth tissues have poor nourishment, which, in relation to the high blood glucose levels, promotes the colonization of overdeveloped microflora in the oral cavity [22]. The presence of PAS material deposition in gingival vessel can be considered as an index of severe diabetic damage [9]. Therefore, slow flow of nutrients and high infection liability promote the destruction process of the periodondium [23].

An important injury that leads to severe handicap in diabetic patients is the development of retinopathy that usually leads to blindness [24]. Most recently, it has been proven that the risk of proliferative diabetic retinopathy was higher in the presence of the periodontal disease [25]. In addition, the occurrence of neuropathy in long-term type 2 diabetes is related to tooth loss and tempomandibular joint disfunction [26].

Therefore, the investigation of the surrounding oral cavity tissues in diabetic patients can demonstrate changes or signs that may alert the physician to control or prevent the development of diabetes mellitus. Furthermore, histological gingival analysis may be routinely utilized for the control of the diabetic disease and perhaps may be considered as a diagnostic method of the severity of the disease.

## Figures and Tables

**Figure 3:**
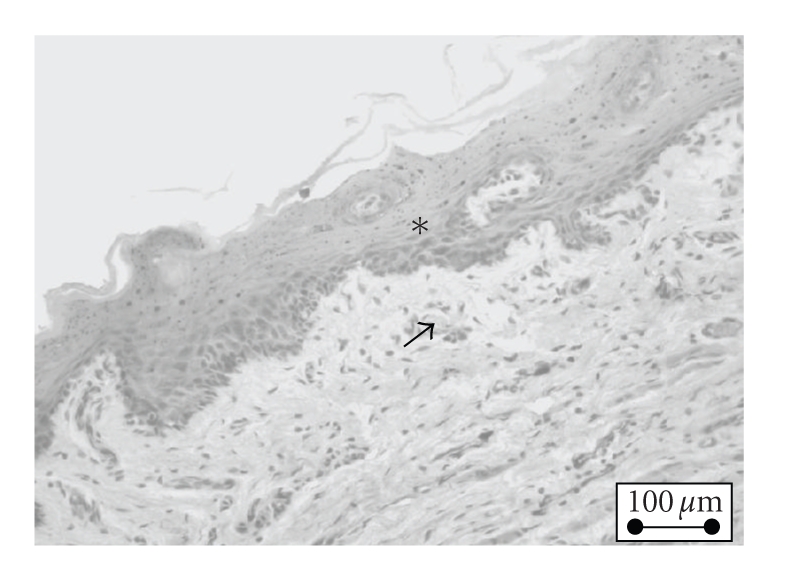
(∗) Normal gingival mucosa. normal squamous epithelium, (↑) normal propria vasculature, and absence of inflammation.

**Table 1:** Clinical indices and laboratory findings upon diabetes mellitus induction.

	Control animals	Streptozotocin-injected animals
Daily food intake (g)	14,86 ± 5,09	19,25 ± 0,95**
Body weight (sacrifice day) g	190 ± 17	160 ± 20**
Hb A1c (%)	5,5%	8,2 ± 1,4%**
Serum glucose mg/dL	90	250**

**P < ,001 versus control.

**Table 2:** Histological findings.

Margin gingival incisor area	Diabetes mellitus	Control
Inflammation	+++	+
Neoangiogenesis	+++	+
Vessel wall thickness	+++	+

Buccal/lingal gingiva		

Hyperkeratosis	+++	+
Epithelium hypertrophy	++	—
